# Supplementary material for: Analysis of PPARγ Signaling Activity in Psoriasis
Source: Int J Mol Sci. 2021 Aug 10;22(16):8603. doi: 10.3390/ijms22168603 (PMC8395241; doi:10.3390/ijms22168603)
Supplement: Supplementary file 1 [file ijms-22-08603-s001.zip › Supplemental materials_Analysis of PPARg signaling activity in psoriasis/Pathway models/Models images and html files/PPARG expressed in cells assotiated with psoriasis/225642.html]

keratinocyte


# CellType keratinocyte

|  |  |
| --- | --- |
| URN | urn:agi-ncimcelltype:C0022567 |
| Total Entities | 2 |
| Connectivity | 6081 |
| Name | keratinocyte |

---

|  |  |
| --- | --- |
| ChildConcepts | prickle cell |
|  | corneocyte |

---

|  |  |
| --- | --- |
| Pathway | EGFR -> Expression Targets in Skin |
|  | ITGB1 -> Expression Targets in Skin |
|  | Elevated Receptors -> Expression Targets in Skin |
|  | NOTCH -> Expression Targets in Skin |
|  | PPARG expressed in cells assotiated with psoriasis |
|  | Neighbors of keratinocyte |
|  | Figure 3\_Mixed network of dexamethasone targets |
|  | Figure 3\_Mixed network of dexamethasone targets |
|  | Overview. Diseases of the skin (chapter 11) |

---

|  |  |
| --- | --- |
| MedScan ID | 10000138 |

---

|  |  |
| --- | --- |
| Alias | keratinocyte |
|  | keratinocytes |
|  | malpighian cells |
|  | keratinized cells of epidermis |
|  | keratinocytic |
|  | cornified cell |
|  | malpighian cell |
|  | keratinized cell of epidermis |
|  | cornified cells |

---

|  |  |
| --- | --- |
| NCIm ID | C0022567 |

---
